# Wheelchair interventions, services and provision for disabled children: a mixed-method systematic review and conceptual framework

**DOI:** 10.1186/1472-6963-14-309

**Published:** 2014-07-17

**Authors:** Nathan Bray, Jane Noyes, Rhiannon T Edwards, Nigel Harris

**Affiliations:** 1 Centre for Health Economics and Medicines Evaluation, Bangor University, Ardudwy Building, Normal Site, Bangor University, Bangor, Gwynedd LL57 2PZ, UK; 2 Centre for Health-Related Research, Fron Heulog, Bangor University, Bangor, Gwynedd LL57 2EF, UK; 3 DesignAbility, Bath Institute of Medical Engineering, The Wolfson Centre, Royal United Hospital, Bath BA1 3NG, UK

**Keywords:** Wheelchair, Childhood disability, Assistive mobility technology, Children, Powered wheelchair, Systematic review, Conceptual framework, Health economics

## Abstract

**Background:**

Wheelchairs for disabled children (≤18 years) can provide health, developmental and social benefits. World Health Organisation and United Kingdom Government reports demonstrate the need for improved access to wheelchairs both locally and internationally. The use of health economics within this field is lacking. Provision of wheelchairs based on cost-effectiveness evidence is not currently possible. We conducted the first systematic review in this field to incorporate evidence of effectiveness, service user perspectives, policy intentions and cost-effectiveness in order to develop a conceptual framework to inform future research and service development.

**Methods:**

We used an adapted EPPI-Centre mixed-method systematic review design with narrative summary, thematic and narrative synthesis. 11 databases were searched. Studies were appraised for quality using one of seven appropriate tools. A conceptual framework was developed from synthesised evidence.

**Results:**

22 studies and 14 policies/guidelines were included. Powered wheelchairs appear to offer benefits in reduced need for caregiver assistance; improved communicative, personal-social and cognitive development; and improved mobility function and independent movement. From 14 months of age children can learn some degree of powered wheelchair driving competence. However, effectiveness evidence was limited and low quality. Children and parents placed emphasis on improving social skill and independence. Participation in wider society and development of meaningful relationships were key desired outcomes. Policy intentions and aspirations are in line with the perspectives of children and parents, although translation of policy recommendations into practice is lacking.

**Conclusions:**

There is a distinct lack of high quality effectiveness and economic evidence in this field. Social and health needs should be seen as equally important when assessing the mobility needs of disabled children. Disabled children and parents placed highest priority on independence and psychosocial outcomes of wheelchair interventions. Translation of policy and guidelines into practice is lacking and more effective implementation strategies are required to improve services and outcomes. Future research should focus on outcome measure development, developing economic evaluation tools and incorporating these into high quality studies to address known research gaps. The novel conceptual framework maps current gaps in evidence and outlines areas for development.

## Background

### Global context

It is estimated that between 10% [1] and 15% [2] of the world’s population is disabled. 10% of disabled people require a wheelchair to provide essential mobility assistance [3]. Approximately 20 million people worldwide do not have access to adequate wheelchairs to maintain mobility and independence, particularly in low-income countries [4]. At present there is inadequate evidence to facilitate appropriate wheelchair service provision and support for those with disabilities [2]. This relates to both understanding of intervention cost-effectiveness and estimates of disability prevalence.

Independent mobility for disabled people and provision of assistive mobility technology (such as wheelchairs) to facilitate this is considered a human right, which calls for all countries to ensure that disabled people are able to access essential assistive mobility technology to promote mobility and independence [5]. Without adequate wheelchairs many disabled people are caught in a cycle of poverty and depravation, lacking the ability to access education, work and social facilities [4]. These issues also have national economic impacts due to loss of productivity and health service resource use [2].

Approximately 5% of children worldwide (around 95 million children aged 14 or under) have a disability [6]. Each disabled child with a mobility impairment has different needs in terms of assistive mobility technology and seating, including consideration of posture, pelvic support and head/neck support. For instance, children with cerebral palsy have the highest demand for specialised seating [7]. Wheelchairs provide essential mobility; it is imperative that they can be used in all places they are required (e.g. school, home and leisure facilities) [8] and that they support the holistic needs of each individual [9]. United Kingdom (UK) and World Health Organisation (WHO) policy states that disabling barriers must be addressed in order to limit exclusion of disabled children from education, healthcare, housing and leisure [8,10].

Appropriate wheelchair interventions are therefore a global imperative in order to reduce disability discrimination and promote equality.

### NHS Wheelchair Services for disabled children in the United Kingdom

There are an estimated 770,000 children and young people under the age of 16 in the UK living with a disability [10]. Several UK government and not for profit organisation (NFPO) reports have found that wheelchair services for children and young people in the UK need improvement in order to meet service user needs [11-15]. These reports reflect the need for a better understanding of the relationship between UK National Health Service (NHS) wheelchair services, effectiveness evidence, service user perspectives and policy intentions.

### Why is a systematic review needed?

Wheelchair interventions can have a range of positive impacts on the lives and health of disabled children and young people. In order to promote effective and equitable wheelchair services both in the UK and globally, better understanding of the effectiveness and cost-effectiveness of wheelchair interventions is needed. Likewise, the opinions of young wheelchair users and their families need to be taken into account to shape services. Social theories of disability state that disability exists as both a physical and social issue. Discrimination and positivist approaches to disability management pose more threat to equality than actual physical impairment [16].

Health economics can play a specific role in the development of wheelchair services by providing essential data on the cost-effectiveness of different wheelchair interventions in both developing and developed countries. This would in theory facilitate better use of resources and greater coverage of services. At present the health economics toolbox is particularly poor when applied to disabilities and children. Development of health economics methodologies based on a social model of health would promote holistic evaluation of effectiveness and cost-effectiveness.

In order to develop an appropriate set of economic tools it is important to explore existing effectiveness, service user opinion and economic evidence. The development of a conceptual framework from synthesised evidence could then be used to guide wheelchair service development in an evidence-based manner. No existing systematic reviews which address these important issues were found prior to conducting this review.

To maintain clarity, we will define a number of key concepts and definitions as follows: the term “wheelchair service” is used to define any private, state or NFPO run service supplying wheelchairs to disabled people based upon assessment of mobility needs by a qualified professional. The term “wheelchair provision” is used to define the supply of a wheelchair intervention to a disabled person by a wheelchair service (as defined above). The term “wheelchair intervention” is used to define any wheelchair supplied to a disabled person by a wheelchair service (as defined above). The term “effectiveness” refers to all relevant clinical and non-clinical outcomes related to wheelchair use, such as (but not restricted to): cognitive, physical and behavioural development; functional mobility and motor skills; independence; educational achievement; social interaction; initiative development; physical and/or emotional well-being; and health-related quality of life. We do not use “effectiveness” to refer to biomechanical outcomes, such as propulsion patterns.

### Aims and objectives

The overarching aim was to explore current effectiveness evidence, service user perspectives, policy and cost-effectiveness evidence in order to develop a conceptual framework to inform future research and wheelchair service development in the UK, with international implications. Four objectives were developed to inform searching, management and interpretation of evidence:

• to determine the effectiveness and cost-effectiveness of wheelchairs for disabled children and young people

• to better understand service user, parent and professional perspectives regarding wheelchairs for disabled children and young people

• to explore current UK policy, NFPO publication and clinical guideline recommendations and intentions regarding wheelchair provision for disabled children and young people

• to determine if disabled children’s desired outcomes match with existing policy aspirations and effectiveness evidence

### Review and synthesis questions

**Figure 1:**
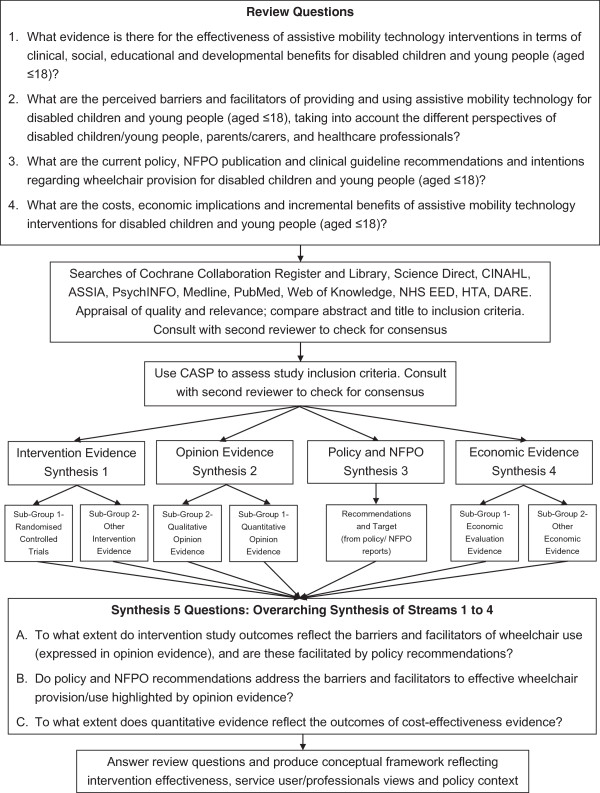
**Systematic review design flowchart.** Flowchart showing the progression of the systematic review from review questions to syntheses. Evidence was streamed by type of evidence and synthesis carried out separately for each stream. An overarching synthesis of all streams brought the different evidence together to build a conceptual framework.

Review questions were formulated for each of the different aspects of this review, with additional questions developed to guide the overarching synthesis of evidence. See Figure 1 for a full list of review questions.

## Methods

### Design

An initial scoping search of the literature was conducted to refine the review scope, processes and keywords. A variety of quantitative, qualitative and policy literature was found, demonstrating the multi-faceted nature of wheelchair interventions. It was therefore decided that a mixed-method systematic review would be the most appropriate way to address the issues of interest. The review questions and a protocol were then developed to guide the review. Searches were conducted between January and April 2012.

The review followed the University of York Centre for Research and Dissemination (York CRD) principles for conducting searches and extracting data [17]. A thematic synthesis approach was used to synthesise qualitative data, informed by the work of Thomas and Harden (2008) [18], while narrative summary was used to synthesise evidence within the intervention, policy and economic streams of evidence [17]. Narrative synthesis was used for the overarching synthesis of different types of evidence [17,19].

An adapted Evidence for Policy and Practice Information and Co-ordinating Centre (EPPI-centre) design and methodology for mixed-method evidence [20] was used to synthesise diverse evidence. Evidence was streamed by evidence and methodology type and results were then synthesised across the streams in a final overarching synthesis (see Figure 1).

A full audit trail was recorded during each stage of the review to enable replicable methods and outcomes. During the screening process each study was screened independently by two reviewers. The second reviewer extracted data and appraised the quality of a selection of intervention studies (n = 5). The findings showed general consensus between the two reviewers, although full appraisal of inter-rater reliability was not conducted due to time constraints.

### Search methods

Internet reference database searching was the main strategy for gathering studies. Inclusion and exclusion criteria were used to refine searches. Databases searched were Cochrane Collaboration Register and Library, Science Direct, CINAHL, Medline, ASSIA, PsychINFO, PubMed, Web of Science, DARE, NHS EED and HTA.

As wheelchair interventions have developed significantly in recent times it was deemed appropriate to restrict the intervention, opinion and economic literature searches to the last 15 years (February 1997 to February 2012). Reference list and hand-searching supplemented electronic searching. Grey literature was also included to limit publication bias. Due to limited translation resources, only studies written or translated into English (UK and international) were considered for inclusion. Search results were managed using the online bibliographic management program *RefWorks.*

Policy and NFPO literature was not available on academic databases. It was identified through internet search engines (Google, Google Scholar), Department of Health/relevant NFPO websites and through hand-searching. Only UK policy/NFPO literature from the last 10 years (March 2002 to March 2012) was considered for inclusion to avoid obsolete literature being included in the review. Although international literature was included in the other streams, it was deemed too expansive to include all international policy in this review. Nonetheless, UK policy is evidence-based including international evidence.

Search terms and keywords were a mixture of Medical Subject Heading (MeSH) and non-MeSH terms. A full list of search terms/keywords can be seen in Table 1, and an example search strategy can be seen in Table 2. In order to ensure that all relevant studies were identified, intervention/opinion evidence search terms were divided into three groups: ‘population’, ‘disability’ and ‘intervention’ (see Table 1). For the economic evidence searches an additional search term group was added: ‘study type/outcomes measures’. As the aim of the mixed-method search was to find all relevant evidence, regardless of study type/outcomes, it was not considered necessary to define the types of studies and outcomes eligible for inclusion. The searches were designed to be sensitive rather than specific. Testing of search terms in the initial scoping searches was used to refine search terms and to test sensitivity prior to starting the full review.

**Table 1:** Keywords for intervention, opinion and economic evidence searches

**Population**	**Disability**	**Intervention**	**Study type/outcome measures (economic evidence searches only)**
Child*	Disab*	Wheelchair	Cost benefit
Adolescen*	Physically impair*	Buggy	Cost utility
Young*	Physical impair*	Mobility technolog*	Cost effective*
Teen*	Handicap*	Mobility aid	Qaly
Disab* child*	Dystroph*	Powered wheelchair	Quality-adjusted life year
Disab*	Cerebral palsy	Mobility equipment	Quality adjusted life year
Adolescen*	Spina bifida	Motorised	Health economic*
Disab* young*	Wheelchair*	Mobility training	Economic analys*
Disab* teen*	Special needs	Wheelchair service	Cost minimisation
	Amputee	Electric scooter	Health care cost*
	Complex needs	Pushchair	Healthcare cost*
	Brain injury	Mobility	Social economic*
	Brain damage*		Social care economic*

*Indicates truncation of keywords.

**Table 2:** Example database search strategies

**Database**	**Search strategy**
**CINAHL and MEDLINE**	Abstract only, 1997–2012 AB ( child* OR adolescen* OR young* OR teen* ) AND AB ( disab* OR physically impair* OR physical impair* OR handicap* OR dystroph* OR cerebral palsy OR spina bifida OR wheelchair* OR special needs OR amputee OR complex needs OR brain injury OR brain damage* ) AND AB ( wheelchair OR buggy OR mobility technolog* OR mobility aid OR powered wheelchair OR mobility equipment OR motorised OR mobility training OR wheelchair service OR electric scooter OR pushchair OR mobility NOT crutch* NOT prosthe*)
**ASSIA**	1997-2012 all(child* OR adolescen* OR young* OR teen*) AND all(disab* OR physically impair* OR physical impair* OR handicap* OR dystrophy* OR cerebral palsy OR spina bifida OR wheelchair* OR special needs OR amputee OR complex needs OR brain injury OR brain damage*) AND all(wheelchair OR buggy OR mobility technology* OR mobility aid OR powered wheelchair OR mobility equipment OR motorised OR mobility training OR wheelchair service OR electric scooter OR pushchair OR mobility) AND all(cost benefit OR cost utility OR cost effective* OR qaly OR quality-adjusted life year OR quality adjusted life year OR health economic* OR economic analys* OR cost minimisation OR health care cost* OR healthcare cost* OR social economic* OR social care economic*)

*Indicates truncation of keywords.

Searches focused on manual and powered wheelchairs specifically due to the volume of recent inquiries in the UK into wheelchair services, their relatively high cost and the unique benefits they provide to disabled children. Economic evidence searches were carried out separately to the intervention/opinion searches in order increase specificity. A full list of inclusion/exclusion criteria can be found in Table 3, with outcomes of interest specified.

**Table 3:** Study inclusion and exclusion criteria (by review question)

**Review question**	**Inclusion**	**Exclusion**
1	*Participants:* Aged 18 or under with a long-term need for mobility equipment for management of a physical disability	*Participants:* aged over 18, short-term need for mobility equipment (e.g. wheelchair after leg fracture)
	*Interventions:* Powered (independent or parent controlled) and manual wheelchairs, buggies and pushchairs	*Interventions:* crutches/sticks, walking frames, adapted shoes, callipers and prostheses, adaptive seating
	*Outcomes:* All relevant clinical and non-clinical outcomes, including (but not restricted to) improved cognitive, physical or behavioural development, improved motor skills, independence, educational achievement, social interaction, initiative development, physical and/or emotional wellbeing and health-related quality of life	*Outcomes:* All outcomes not stated in inclusion criteria
	*Evidence:* All effectiveness evidence related to effectiveness of assistive mobility technology including randomised controlled trials, quasi-experimental trials, clinical trials, epidemiological research, cohort studies, non-randomised controlled trials, mixed-method research, systematic reviews and survey data.	*Paper details:* Not written or translated into English, published over 15 years ago
2	*Participants:* Children/young people aged 18 or under with a long-term need for mobility equipment for management of physical disability, parent/carer of a child or young person aged 18 or under with a long-term need for mobility equipment for management of a physical disability, healthcare professionals treating/rehabilitating children/young people aged 18 or under with a long-term need for mobility equipment for management of a physical disability	*Participants:* children/young people and parents/carers/healthcare professionals of people aged over 18, short-term need for mobility equipment (e.g. wheelchair after leg fracture)
	*Interventions:* Powered (independent or parent controlled) and manual wheelchairs, buggies and pushchairs	*Interventions:* crutches/sticks, walking frames, adapted shoes, callipers & prostheses
	*Outcomes:* All experiences, views, perspectives, thoughts and feelings of children/young people, parents and healthcare professionals towards mobility equipment and provision	*Outcomes:* All outcomes unrelated to barriers, facilitators, positives and negatives of mobility equipment provision
	*Evidence:* All studies using qualitative methodologies, including ethnographic research, grounded theory research, case studies, phenomenological research, qualitative systematic reviews, meta-ethnography, mixed-method research and survey data.	*Paper details:* Not written or translated into English, published over 15 years ago
3	*‘Audience:* Children/young people aged 18 or under with a long-term need for mobility equipment for management of physical disability, parent/carer of a child or young person aged 18 or under with a long-term need for mobility equipment for management of a physical disability, healthcare professionals treating/rehabilitating children/young people aged 18 or under with a long-term need for mobility equipment for management of a physical disability, decision and policymakers influencing NHS wheelchair services	*Audience:* children/young people and parents/carers/healthcare professionals of people aged over 18, service users with short-term need for mobility equipment (e.g. wheelchair after leg fracture)
	*Publications:* All policy, guidelines, frameworks and government and third sector publications regarding mobility equipment provision, use, maintenance and funding	*Publications:* Policy and guidelines from outside of United Kingdom, Obsolete or out-of-date policies and guideline, published over 10 years ago
4	*Participants:* Aged 18 or under with a long-term need for mobility equipment for management of a physical disability	*Participants:* aged over 18, short-term need for mobility equipment (e.g. wheelchair after leg fracture)
	*Interventions:* Powered (independent or parent controlled) and manual wheelchairs, buggies and pushchairs	*Interventions:* crutches/sticks, walking frames, adapted shoes, callipers and prostheses
	*Outcomes:* All relevant clinical and non-clinical outcomes, including (but not restricted to) improved cognitive, physical or behavioural development, improved motor skills, independence, educational achievement, social interaction, initiative development, physical and/or emotional well-being and health-related quality of life. Direct and indirect costs, impacts on quality-adjusted life years gained, utility scores, quality of life measures and incremental cost-effectiveness will inform the economic outcomes.	*Outcomes:* All outcomes not stated in inclusion criteria
	*Evidence:* All economic evidence related to assistive mobility technology including cost-benefit, cost-utility and cost-effectiveness analyses. Partial economic evaluations (including cost analyses, cost-description studies and cost-outcome descriptions) will also be included. Economic evaluations conducted alongside RCTs, quasi-experimental trials, clinical trials, epidemiological research, cohort studies and non-randomised controlled trials will all be considered	*Paper details:* Not written or translated into English, published over 15 years ago

### Screening

Three stages of screening were used. For the initial screening process all identified study titles were assessed for relevance against the inclusion/exclusion criteria. A second screening process was used to assess relevance of studies by their abstract. When relevance was unclear the full study was obtained and reviewed. All studies that were considered relevant after initial and second screening were obtained in full and underwent a final screening process. In order to reduce bias a second researcher reviewed each study independently and consensus was reached regarding inclusion. A formal screening process was not required for the policy literature, as searches were conducted using search engines and searching of government and NFPO websites. Searching stopped once saturation had been reached and no new policy/guideline reports were found.

### Data abstraction

Basic information (author, publication year, title) was collected for all studies. Additionally, evidence specific data extraction tools were made for the purpose of extracting appropriate findings from the different types of literature. Each tool was tailor made for a specific type of evidence, which allowed the extraction of data to be specific to each stream of evidence. See Table 4 for a full list of data extraction criteria by evidence type.

**Table 4:** Data extraction criteria by evidence type

**Intervention evidence**	Aims, objectives, hypotheses, study type, methodology, randomisation details, number of groups, number in each group, number completed in each group, data collection time points, participant characteristics, participant age range, type of intervention(s), inclusion/exclusion criteria, country/ethnicity, baseline characteristics, content of intervention(s), duration of intervention(s), control intervention(s), follow-up period, outcomes and measures, narrative summary of findings (including statistical significance, confidence intervals and effect size), identified themes/concepts.
**Opinion evidence**	Aims, objectives, hypotheses, study type, methodology, number of study groups, number in each group, number completed in each group, data collection time points, participant characteristics, participant age range, type of intervention(s), inclusion/exclusion criteria, country/ethnicity, follow-up period, narrative summary of findings, identified themes/concepts.
**Policy/NFPO literature**	Type of publication, topic, aims, objectives, related conditions and disabilities, age range of affected individuals/target audience, related interventions, narrative summary of recommendations and guidance.
**Economic evidence**	Perspective, aims, objectives, hypotheses, study type/methodology, price year/currency, randomisation details, number of groups, number in each group, number completed in each group, data collection time points, measure of benefit, participant characteristics, participant age range, type of intervention(s), inclusion/exclusion criteria, country/ethnicity, baseline characteristics, content of intervention(s), duration of intervention(s), control intervention(s), follow-up period, outcomes and measures, narrative summary of findings (including statistical significance, confidence intervals and effect size), identified economic costs and implications, cost per QALY/Incremental cost-effectiveness ratio conclusions, inflated (2012) cost per QALY/Incremental cost-effectiveness ratio conclusions.

Summary measures could not be used across the intervention evidence due to differences in sample demographics, outcome measures and interventions (see Additional file 1).

### Evidence synthesis

Evidence was divided into four streams according to methodology and topic to enable separate syntheses by evidence type (see Figure 1):

1. Intervention Evidence: all quantitative studies determining the effectiveness and outcomes of relevant interventions.

2. Opinion Evidence: all studies exploring perspectives and views relating to relevant interventions in childhood disability.

3. Policy and NFPO Literature: all relevant policy, NFPO and clinical guideline literature.

4. Economic Evidence: all relevant economic and cost-effectiveness evidence.

Intervention and economic streams were not synthesised due to vast differences in studies and lack of statistical evidence within each stream (see Additional file 1 for further details), thus narrative summary was conducted. Intervention evidence outcomes were grouped by type.

For the qualitative opinion evidence, thematic synthesis [18] was conducted in order to identify key themes of service user and professional perspectives on wheelchair provision and interventions. This process included three stages:

1. Line-by-line coding of findings to order the findings into initial codes

2. Grouping of initial codes to form broader descriptive themes

3. An overarching synthesis of the descriptive themes to create higher-level analytical themes.

Survey data that could be coded (such as open-ended questions) was incorporated into the thematic synthesis. For survey evidence that could not be line-by-line coded, narrative summary was used to form a structured narrative of results. These data were later synthesised with the thematic synthesis findings and incorporated into the appropriate descriptive themes.

A final over-arching narrative synthesis was undertaken to draw together the results across the different streams of evidence. The framework developed by Oliver et al. [19] was used to structure this synthesis and compare results across streams of evidence. To facilitate this three over-arching questions were developed (see Figure 1).

### Conceptual framework development

A conceptual framework for developing cost-effective wheelchair services for children and young people was refined from the overarching synthesis of evidence. Findings from the different streams of evidence were interrogated, discussed, mapped, charted and refined through further discussion within the research team to build a deeper understanding. The most important findings were selected and integrated into a conceptual diagram. A programme theory for an evidence-based pathway through wheelchair services was developed which highlighted gaps in knowledge and current services.

## Results

### Search and screening outcomes

**Figure 2:**
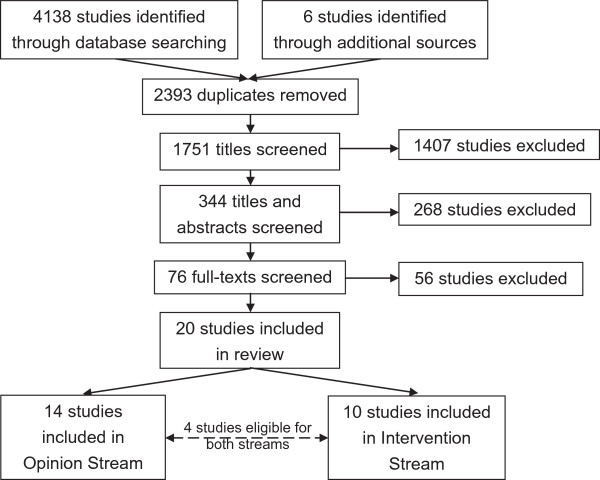
**Search results for intervention and opinion evidence searches.** Flowchart showing the search results and screening stages of the intervention/opinion evidence searches.

**Figure 3:**
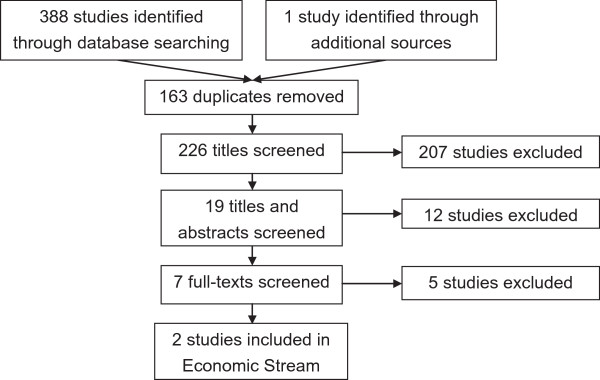
**Search results for economic evidence.** Flowchart showing the search results and screening stages of the economic evidence searches.

A full list of included studies can be found in Additional file 1. See Figures 2 and 3 for the screening process outcomes. In total 4144 studies were found in the intervention/opinion evidence searches, of which 2393 duplicates were removed (see Figure 2). After screening titles and abstracts, 76 full-texts were left. In total a further 56 were excluded after screening of full-texts, leaving 20 deemed eligible for inclusion: 10 in the intervention evidence stream and 14 in the opinion evidence stream (four studies were eligible for both streams of evidence). Reasons for exclusion included focus on adults (or inability to extract child data), lack of primary data and focus on biomechanical outcomes.In total 389 studies were found in the economic evidence searches, of which 163 duplicates were removed (see Figure 3). After screening titles and abstracts, seven full-texts were left. In total two were deemed eligible for inclusion. Reasons for exclusion included focus on adults and lack of primary data. In total 14 policy and NFPO reports were deemed eligible for inclusion.

Evidence could not be grouped and analysed using meta-analysis due to heterogeneity of samples, methodology, interventions and outcomes (see Additional file 1). Summary outcomes and synthesis of statistical data were also inappropriate due to heterogeneity. Narrative summary was conducted to form a structured narrative of the results.

### Intervention evidence

10 studies explored the effectiveness of wheelchairs for children with disabilities: Seven determined the effectiveness of powered wheelchairs (PWC) [21-27]; one compared ultralight and lightweight wheelchairs [28]; and two looked generally at the impact of assistive devices/environmental modifications (including PWCs) [29,30].

Of the 10 studies, five looked specifically at children with cerebral palsy and orthopaedic disabilities [22,24,25,27,29]. The remainder included children with a range of disabilities such as complex developmental delay [26], spinal muscular atrophy [21], spina bifida [28] and motor impairment preventing functional independent mobility (including conditions such as cerebral palsy) [23,30]. Child participant ages ranged from 14 months to 12 years.

Only one randomised controlled trial (RCT) was found [23]. The remaining studies used a range of methodologies, including case study, case series, quasi-experimental design, ‘A-B-A’ single subject design, single-subject withdrawal design and cross-sectional survey.

Only four studies employed statistical analysis and sample sizes were small; several studies used a single case-study design. The single RCT [23] was of moderate quality and had a small sample size of 28 (equally split between intervention and control groups). Outcome measures used within each study are presented in Additional file 1*.*

Quality appraisal of studies indicated that they were generally low quality (see Additional file 2). Risk of bias was assessed as part of the critical appraisal outcomes (see Additional file 2). The intervention evidence results are therefore presented with caution, taking into account the quality of results and potential risk of bias.

The vast majority of the evidence was in reference to PWCs, and thus wider understanding of wheelchair effectiveness in general is not possible. There was some evidence to indicate that ultralight wheelchairs are preferable to lightweight chairs in terms of ease of propulsion [28]*.*

The intervention findings were grouped by type of benefit and categorised accordingly through narrative summary. Statistical significance is presented where reported. The emergent categories of benefit were:

1. Caregiver assistance and benefits

PWCs reduce need for caregiver assistance [21,23,29] and reduce caregiver stress [24]. PWCs have statistically significant effects on need for caregiver assistance for mobility (p = .01, ES = 12.35 [6.5-20.5] at 90% CI) and self-care (p = .0007, ES = 11.95 [7.5-16.15] at 90% CI) [23].

2. Social and play skills

For children with orthopaedic disabilities aged 18 to 72 months PWCs significantly positively affect: pro-social adaptive social behaviour (F = 5.30, p < .05 at 95% Confidence Interval[CI]); interactions with family (F = 3.2, p < .05 at 95% CI); indoor play motor activities (F = 4.53, p < .05 at 95% CI); quality of interactive play (F = 4.24, p < .05 at 95% CI); and developmental level of symbolic play (F = 4.9, p < .05 at 95% CI) [22].

For children with orthopaedic disabilities aged 18 to 42 months PWCs also facilitate significant improvements in: interactions with family (F[2,21] = 3.3, p < .05); parental satisfaction with child’s social and play skills (F[2,21] = 3.27, p < .05); and parents’ belief that the general public accepts their child (F[2,21] = 3.65, p < .04) [24].

3. Functional movement and mobility

PWCs improve functional mobility [21,23] and child-initiated movement [26], with significant impacts on mobility functional skill (p = .04, ES = 6.5 [2-11] at 90% CI) [23] and parental satisfaction with child’s ability to go where they desire (F[2,21] = 11.69, p < .05) [24].

4. Developmental benefits

PWCs potentially offer developmental benefits in: communication, cognition and personal-social domains [21]; receptive communication skills [23]; and occupational performance [25]. PWCs can significantly improve: activities of daily life (in the dimension of functional limitation) (p < 0.00001) [25]; receptive communication (p = .03, Effect size = 6.1 [0.95-9.2] at 90% CI) [23]; and overall development scores (p = .083, ES = 2.0 [0.0-3.5] at 90% CI) [23].

5. Driving skill and competence

Children as young as 14 months can learn some degree of PWC driving competence [27]. PWC driving competence improves after six to eight months of use (p < 0.01) for children with cerebral palsy aged three to eight years [25].

### Opinion evidence

14 studies explored the experiences and perspectives of young wheelchair users, their parents/carers and related professionals (e.g. clinicians, teachers, therapists). Seven studies were related specifically to PWCs [24,31-36] and six were related to both manual wheelchairs and PWCs [29,30,37-40]. The majority of studies explored physical disabilities generally in children using wheelchairs (manual and/or powered), although four of the studies looked specifically at children with cerebral palsy and orthopaedic disabilities [24,29,38,39].

Most of the participants were families of disabled children (child age range from 18 months to 18 years), although four studies also included professional participants (e.g. wheelchair suppliers, teaching staff, therapists, clinicians) [32,33,35,39] and four directly included the opinions of disabled children and young people [36-38,40]. Five studies used qualitative methodologies exclusively (including phenomenology and grounded theory) [31,32,37,38,40], while the rest used either questionnaire/telephone survey (with quantitative and qualitative data), retrospective research or cross-sectional research.

12 descriptive themes were generated from line-by-line coding of the evidence (see Table 5), which were then synthesised to make higher-order analytical themes. Analytical theme generation was focussed on PWCs due to the focus of the qualitative evidence. Making broader assumptions about other forms of assistive mobility technology (e.g. manual wheelchairs and pushchairs) would have been inappropriate due to the lack of evidence. In total, five analytical themes were developed:

**Table 5:** Descriptive themes generated from opinion evidence

**Descriptive themes**	**Examples**
Wheelchair services	Providers, repair and maintenance
Environmental factors	Home, public and school environment
Chair characteristics	Size, weight and usability
Individual ability	Health, physical and developmental readiness
Family factors	Attitude, support and finances
Safety of use	Build quality, accidents and safe use
Learning to use wheelchair	Learning mobility and wheelchair safety
Social factors	Socialisation, participation and others’ attitudes
Quality of Life	Self-esteem, confidence and well-being
Physical factors	Comfort, support and positioning
Independence	Freedom and independent movement
Developmental impact	Attaining milestones

1. Wheelchair services do not consistently meet all needs of service users, and parents are resigned to this

Specific wheelchair service issues included long waiting times [37,38], poor maintenance procedures [34,37,38], strict eligibility criteria [34] and differing opinions of needs [31,36]. There appeared to be consensus that services were doing the best they could, and thus there was resignation to current standards of provision [37].

Participants highlighted issues around lack of information provision with regards to choice of wheelchairs, potential wheelchair benefits and funding available to families [34,38,39]. The evidence highlighted the financial burden placed on families having to pay for their own essential wheelchairs, maintenance and adaptations [34,36,38].

2. Parents find it difficult to accept their child’s need for a wheelchair

There was a perception that accepting a wheelchair was an admission that independent mobility without a wheelchair would never be possible [31]. The process of coming to terms with both manual wheelchair and PWC use for their children was long, and for many parents the perception of wheelchairs was negative before their child had used one [31,33]. Results indicated that 84% (of n = 25) of parents did not accept the idea of PWC before provision, but 92% (of n = 25) had positive feelings after PWC provision [17]. This demonstrates that a process of adjustment is required. 23% (of n = 140) of wheelchair clinicians and suppliers felt that a lack of family support limited wheelchair provision [33].

3. PWCs are a tool for independence and socialisation

PWC use facilitates development of independence in disabled children [25,31,34,38], and this independence subsequently allows greater socialisation [37]. It was found that the use of a PWC had a positive effect on the attitudes of others [31,34] with people seeing the child as an independent ‘whole person’ [31]. This change in the attitudes of others allowed further socialisation, age-appropriate activities and acceptance by peers and other people in the community [31].

4. Wheelchairs offer a new lifestyle to disabled children and their families

Wheelchairs were perceived to offer a new lifestyle for disabled children and their families [25,30,31,34,37]. PWCs were believed to provide improvements to quality of life (over no wheelchair equipment and manual wheelchairs) [24,34]; ability to take part in age-appropriate activities and responsibilities [31,37]; and overall freedom [36]. After PWC provision children were able to socialise more [31,34,36-38]; to integrate better into school and community settings [37]; and were less reliant on the help of others [31]. Parents acting as ‘responsive partners’ facilitate learning to use a PWC [32].

5. Structural and environmental factors are a major barrier to the use of wheelchairs

Poor access to buildings [29,31,36,38], difficulty transporting wheelchairs [24,29,31,33,36,37,39] and poor disabled parking facilities [36,38] were identified barriers to wheelchair use. Community and social environments were reported to often be unfit for wheelchair access [31,36-38]. The size and bulk of wheelchairs was reported to inhibit integration with other peers as well as affecting use and transport [38,40].

### Policy and guidelines

14 policy and NFPO reports were included in the review: three were produced by NFPOs [41-43]; 10 were produced by UK government and Department of Health organisations [8,9,11-15,44-46]; and one was a joint publication produced by the UK government and an NFPO [47].

Findings from the policy and NFPO evidence were grouped by type of recommendation/target. Seven emergent categories were identified:

1. Waiting times

The most commonly identified recommendation was reduction of waiting times for assessment, delivery and maintenance of wheelchairs (e.g. maximum of 18 weeks from referral to delivery [41]) [8,9,12-15,41,43,44].

2. Joint-working and multi-agency approach

The need for joined-up working between health, social care, education and NFPOs was a recurrent theme throughout the literature, with a general aim to improve services and to extend the scope of provision [8,9,11,12,14,15,45]. This included pooling of budgets [11] and outsourcing training/tuition [9].

3. Effective use and outcomes

Several publications highlighted the need for wheelchairs to be useable in all places required in order to maximise effectiveness [8,12,15]. There were recommendations for assessment and provision to take into account the holistic needs of service users as part of maximising social, physical and lifestyle outcomes and promoting independence [9,11,12,15,41].

4. Funding and procurement

Recommendations included: ring-fenced budgeting for PWC provision [41]; improved efficiency, productivity and innovations in the NHS wheelchair product line [9];pooling of budgets between health, social care and education authorities [11]; and efficient procurement, long-term cost control and initial investment [47]. Productivity savings should be re-invested into wheelchair and seating provision [9].

5. Aftercare and information

Maintenance and review procedures need attention, with clear and defined minimum standards for reviews [8,13,15]. Better quality information for service users regarding support, additional funding/grants, tuition and local service changes was recommended [8,14].

6. Eligibility criteria and assessment

Comprehensive access to multi-disciplinary assessments was of high priority [14,15,43,45]. There was also recommendation for extended equipment loan programmes [45] and national consensus of eligibility criteria and outcomes [13,46].

7. Service user involvement

Recommendations included: designing services around the needs of service users [9,43,45]; supporting service users to make informed decisions about treatment/care and support [15,44]; and improving communication with users and stakeholders [11].

### Economic evidence

Two eligible studies exploring the cost-effectiveness of wheelchairs for disabled children were found. Due to the lack of evidence and the heterogeneity of data (cost, year, outcomes, interventions etc.) it was not possible to synthesise the findings, therefore narrative summary was conducted.

Neilson et al. [48] found the cost per quality-adjusted life year (QALY) (compared with a ‘do nothing’ scenario) for provision of a powered indoor/outdoor wheelchair ranged from £734 to £1378 (dependent on time horizon) based on a cost per wheelchair intervention ranging from £1500 to £2000. Inflation to 2011 prices [49,50] provides a cost per QALY of £1187 and £2229 (40 and 50 year time horizon respectively). These results indicate that PWC interventions can be cost-effective in relation to the National Institute for Health and Clinical Excellence (NICE) £20,000 to £30,000 intervention cost threshold. Estimates are based on a single subject within the study, whose age is not stated. Costs used to generate QALYs were based on a single intervention over a 40 or 50 year time horizon.

Frontier Economics [51] examined the impact of NFPO (Whizz-Kidz) involvement in the running of NHS Primary Care Trust paediatric wheelchair services. Meeting unmet service demands cost an extra £108,000 and provided an additional 10.7 to 14 QALYs, resulting in a cost per QALY of between £7,700 and £9,800 for meeting unmet service demands. This evidence has not been published by a peer reviewed journal, thus its application in this review is limited.

### Over-arching synthesis

The majority of data were specifically about PWC provision and use, which is reflected in the over-arching synthesis. A number of additional findings were elicited from further synthesis of the entire integrated dataset:

#### ***Higher quality wheelchair services take into account the needs of the whole family***

Intervention and opinion evidence shows that wheelchair provision can be beneficial for both the wheelchair user and their family, including parental independence [30,37]; reduced need for caregiver assistance [21,29,30]; facilitation of positive parental feelings [25]; and reduction in parental stress [24].

As use of a PWC requires family involvement it is important that the home environment and the ability to transport a wheelchair is assessed and facilitated where possible. The cost of maintenance, repairs and adaptations can be prohibitive for families [36,38], thus funding arrangements at policy level should ensure that these costs are covered or available grants are signposted [8,41].

Each service user may benefit from having a clear point of contact for any queries they may have [8,13,15]. Services may be best developed in consultation with children and families to promote child and family-centred services [9,44].

#### ***Disabled children benefit when psychosocial needs are considered alongside health needs***

The psychosocial needs of children using PWCs appear to be of highest priority for service users and their parents [31,34,36-38]. Children are perceived to benefit more when PWC provision takes into account where the wheelchair will be used, and ensures that any supplied PWC is fit for use in all places it is required [8,12,15]. Social benefits of PWCs were found in the intervention evidence, including positive differences in interactions with family after PWC intervention [24] and pro-social adaptive social behaviour [22]. A holistic approach to assessment, with performance measures that consider psychosocial, environmental and lifestyle needs alongside clinical requirements are therefore important [9,11]. Additional benefits and efficiencies were also noted from joined-up working and planning between health, social services and education departments [8,9,11,12,14,15,45].

It is of note that the majority of opinion evidence (n = 9) related to children aged under 14 years. This indicates that there may be a lack of evidence on key periods of transition, such as moving from child to adult services.

#### ***Disabled children could benefit if policy recommendations focussed on services meeting individual needs rather than following strict eligibility criteria***

Inefficiencies (such as long waiting times) need to be reduced [11,37,38] and loan programmes developed to allow children to try wheelchairs before provision [33]. Strict eligibility criteria can be prohibitive to each child receiving the right wheelchair [12,15,34], thus uniform and flexible national eligibility criteria may help to address inequity in services [13,41,46]. Joined-up working between agencies could further enhance services [8,9,11,12,14,15,45].

#### ***Without appropriate outcome measures the holistic benefits of PWC interventions cannot be evaluated***

Evidence of effectiveness and validated clinical practice outcome measures are needed in all aspects of health services [52]. The development of reliable and valid measures of holistic benefits is needed in order to measure the wider benefits of PWC interventions. When appropriate outcome measures are available PWC intervention goals can more easily focus on the tangible benefits of developmental gains rather than just development.

Opinion evidence continually demonstrated the importance of independence and the subsequent perceived benefits to service users and families [25,31,34,38]. Developmental benefits were observed in intervention and opinion evidence [21,23,31,32,38]. Opinion evidence highlighted the potential quality of life benefits of PWCs, including increased happiness, enjoyment of life, motivation, self-confidence and reduced frustration [34], as well as increased dignity and activities of daily living [31].

#### ***Disabled children may benefit more when physical outcomes of PWC use are seen as facilitators to wider holistic benefits, but lack of translation of evidence into practice hinders progress***

The key benefits from provision of wheelchairs for service users and their families were lifestyle oriented, with a focus on social and independence effects. Policy and NFPO literature does take into account these wider benefits; recommendations highlight the need to set minimum standards for wheelchairs that are useable in all places required [8,12,15] and that promote independence [12,41] with measurable outcomes. However the translation of these recommendations into practice is apparently weak.

#### ***Disabled children would benefit from public buildings and spaces that promote inclusion of disabled people***

Policy and NFPO literature states that wheelchairs should be useable in all places they are required [8,12,15], however this appears to be in reference to the wheelchair itself rather than more accessible public places. Poorly designed public spaces restrict children’s ability to participate socially [29,31,36-38,40]. Legally enforced equality of access is therefore likely to improve wider lifestyle benefits of wheelchair users.

Home adaptation with clear advice provided to all service users and families regarding funding and grant entitlements are also important factors that impact on health-related quality of life outcomes [8]. Some families have issues using wheelchairs due to inaccessibility of the home environment [33,39]. Regular review and maintenance procedures can help to ensure that wheelchairs are fit for purpose [13,41].

### Conceptual framework

**Figure 4:**
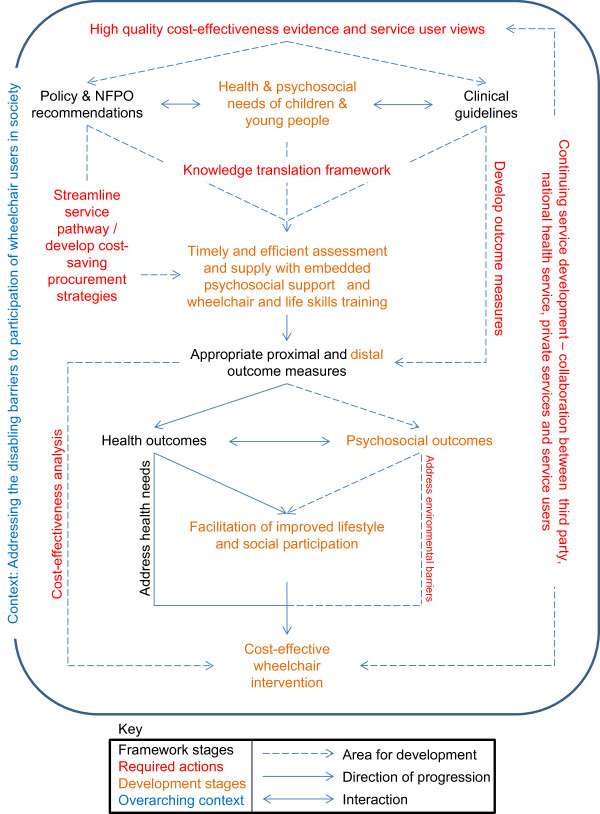
**Conceptual framework for developing cost-effective wheelchair services for children and young people.** A conceptual framework was developed from the overarching synthesis of evidence. It details areas for development and required actions in order to develop cost-effective wheelchair services.

The conceptual framework (see Figure 4) maps how further research and service development can lead to cost-effective wheelchair services and interventions. It details areas that need development and where actions for improving both the effectiveness and cost-effectiveness of wheelchair services for children and young people are required.

Areas for future development include: conducting and making available high quality effectiveness, cost-effectiveness and qualitative evidence; developing a knowledge translation framework; streamlining management and procurement strategies; developing appropriate outcome measures; addressing environmental barriers to wheelchair use; conducting robust cost-effectiveness analyses; and ensuring continued service development with collaboration between third party, NHS, private services and service users (children/young people and their families).

## Discussion

The major contribution to knowledge from this novel mixed-method review comes from the synthesis of diverse evidence to form a new conceptual framework and programme theory for optimal wheelchair service provision for children.

Within this overall context, the most important finding is that for children and young people wheelchairs offer more than mobility; they offer enhanced independence, social integration and participation in age-appropriate activities. It is therefore paramount that wheelchair interventions are seen as facilitators to a new way of life. Nonetheless, disabled children and parents can find the transition to wheelchair use a traumatic process that is not yet sufficiently understood. Being able to individually tailor support for children and parents, and being able to measure these wider lifestyle benefits, is therefore a priority. Further research is needed to address these significant gaps in current knowledge.

UK policy and NFPO recommendations are reflective of the perspectives of young wheelchair users and their families, but there is a lack of effective translation of policy and evidence into practice. Although policy recommendations do correlate with the opinion evidence, the barriers to effective provision and use of wheelchairs have continued to prevail in UK NHS services over many years [11-14]. The key to improving outcomes for children and young people lies in improving service delivery, organisation and translation of knowledge of what works and what children desire from their wheelchairs.

The translation of evidence and knowledge into practice is not simply a case of publishing guidelines and policy. Evidence based practice requires specific action and commitment from services, for instance through the implementation of a knowledge translation framework such as the Knowledge to Action Process [53]. This knowledge translation process recognises the importance of gathering and synthesising evidence in a robust and replicable manner, and emphasises use of appropriate dissemination techniques and effective exchange of knowledge between researchers and knowledge users. This process is particularly useful for areas where research may be lacking, and encourages the synthesis of evidence through systematic reviews and meta-analyses in order to gather and build upon the current knowledge base [53].

Translation of evidence into practice is mitigated by the level of evidence, the context, the presence of facilitation and the success of implementation (assessing organisational outcomes and achievements) [54]. Services must therefore make a commitment to implementing a framework that promotes the translation of evidence into practice. Without specific commitment to change, services are unlikely to be developed in a way that promotes and facilitates positive change in-line with service user needs and evidence of effectiveness.

New tools have been produced to facilitate adoption of evidence into practice that could help identify the problems of evidence translation in local contexts. For example, The NHS Institute for Innovation and Improvement [55] developed the ‘Spread & Adoption’ tool to aid in the assessment of innovation implementation likelihood. The purpose of this tool is to highlight small changes that can be made to promote change and ensure that an organisation is ready to implement new ideas. With the use of tools such as this, organisations can prioritise factors that require action and determine barriers to change and innovation.

The ‘Any Qualified Provider’ principle concerns the tendering process whereby any qualified provider can compete for NHS contracts that are awarded by the Department of Health in England. Approved providers may include state/private hospitals, charities, private organisations and certain retailers (e.g. private wheelchair suppliers). This allows patients to make informed decisions about their healthcare based on service attributes important to them, for instance how geographically close a service is or the quality of care provided. This principle promotes services that are developed around the holistic needs of the service user [56] as they can seek the most appropriate provider for their needs. It has potential for wider application if more evidence of effect is available to help inform decisions. Focussing on integrating agencies to provide better care and services for disabled children is also of paramount importance. Wheelchair services need to think outside the health domain and consider the wider needs of disabled children to ensure they are not excluded from education and social settings.

There is a distinct lack of high-quality effectiveness and cost-effectiveness evidence within this field. Although many studies have used robust methods to explore bio-mechanical impacts of mobility interventions (which were not relevant for this review), these do not reflect the outcomes of services from a carer or service user perspective. The intervention evidence, although limited by quality, demonstrates that wheelchair interventions may have a range of positive effects beyond mobility. However these results should be viewed with caution due to the limited quality of evidence. More evidence is required to understand how effective interventions can be measured and achieved for all service users. This requires studies to use large sample sizes, robust methods and diverse outcome measures.

The application of health economics could enable a better understanding of the cost-effectiveness of wheelchair interventions, and thus benefit service-commissioning and funding allocation, and enable these practices to be evidence-based and equitable. The limited economic evidence in this review may be considered best evidence in the field due to the lack of other research into the cost-effectiveness of wheelchairs for children and young people.

Future research should focus on developing more appropriate outcome measures, health economic methods, and exploring the use of quality of life or capability measures to determine effectiveness from a more holistic perspective. Current wheelchair service outcome measures focus on clinical outcomes and service quality (e.g. QUEST [57]), which do not reflect all of the needs of service users. Incorporation of generic preference-based measures into routine data collection would also allow local and international collection of utility data. This could in turn be used to develop cost per QALY estimates and utility changes facilitated by wheelchair interventions. Furthermore, this evidence would allow comparisons with other healthcare interventions and understanding of incremental cost-effectiveness. This would in turn encourage appropriate funding allocation and provision based on robust effectiveness evidence.

Designing high-quality research in this field has specific challenges, particularly if looking generally at wheelchair interventions across a range of disabilities. Mobility impairment can be as a result of many different conditions, and thus needs and interventions can be highly variable. This has implications for conducting large scale trials using clinical outcomes. Likewise, interventions are likely to be highly variable across different conditions. Health-related quality of life and capability measures would allow a universal outcome that reflects the wider benefits of such interventions, and therefore would be a more appropriate approach to understanding the effectiveness of various interventions.

Although the use of QALYs can be contentious [58], it provides a universal measure that can compare the effectiveness of disparate interventions. For instance, different types of wheelchairs for different types of disabilities could be compared using a single outcome (QALY gains). This data could be collected alongside clinical outcomes in order to encourage holistic interventions that fit in with the needs and desires of young wheelchair users.

At present child and parent proxy versions of validated health-related quality of life measures do exist, for instance the Health Utilities Index (HUI) [59]. However, their relevance for wheelchair users is still to be demonstrated. Some measures, such as the PedsQL, have additional bolt-on questions for particular conditions (such as cerebral palsy) which take into account the condition-specific aspects of quality of life [60].

If wheelchair services in the UK and internationally were to adopt a single set of outcome measures a wealth of data could be generated, which could be used to evaluate the holistic effectiveness of wheelchair interventions for children and young people. This data could be used to aid the development, supply and maintenance of wheelchairs. It would promote interventions that reflect the desires of service users and would allow outcomes to be measured appropriately from the perspective of the service user and the clinician. Furthermore, services could be structured around the needs of service users.

Within a UK healthcare system context, the findings provide impetus for NICE to consider wheelchair services (both adult and child) a high priority. NICE provides national clinical guidelines on healthcare interventions, medication and new health technologies in order to ensure high quality and evidence based care for patients within the NHS [61]. To date NICE have produced little guidance on disability interventions.

### Review limitations

No major deviations from the protocol were noted. In the spirit of transparency, it is worth considering some potential limitations. The original aim was to understand wheelchair interventions more generally, however due to the general focus in the literature on PWC interventions, the findings have greater relevance to PWC. Over half of the intervention studies looked specifically at children with cerebral palsy. Furthermore, the intervention evidence was of low quality and at risk of bias, thus the findings must be viewed with caution.

Although evidence included in this review may not be universally generalizable to all conditions, it still offers a better understanding of what benefits are afforded by wheelchair provision. More research may be needed to see if particular benefits from wheelchairs are universal across conditions.

The lack of economic evidence highlights the issues of applying health economics to wheelchair provision for disabled children and justifies further research within this field. The lack of RCTs in this field highlights the ethical and methodological issues of wheelchair intervention studies in children. However, the study by Jones et al. [23] establishes that an RCT can be a useful and ethically sound approach when conducted appropriately. For instance, it is unethical to withhold wheelchairs from those that require them, thus standard issue wheelchairs could be used in the control group and more technologically advanced equipment in the intervention group. Likewise research examining manual versus powered wheelchairs could utilise a similar RCT setup.

Only evidence written or translated into English was included in this review, which may have excluded valuable research written in other languages.

## Conclusions

Wheelchairs offer varied benefits to disabled children in terms of health, development and social inclusion. At present NHS wheelchair services in the UK are not meeting all of children’s needs and service development is required.

Findings derived from the evidence are relevant for NHS services and have some implications for wheelchair services globally. Wheelchair services have an invaluable role in promoting equality for disabled people. If these services can address disabling barriers for children at a young age, they may be able to facilitate more inclusion in education and society.

There are important gaps in current knowledge regarding health economic methods and available outcome measures, which hinder further service development and research. Health economics has an important role in developing effective, efficient and equitable wheelchair services globally. The lack of economic evidence in this field highlights the lack of appropriate methods to measure cost-effectiveness. Establishing the cost-effectiveness of interventions is a priority to promote efficient services.

Collaboration between countries on future research would allow the collection of a wealth of data regarding intervention effectiveness and cost-effectiveness. The use of universal and validated outcome measures across countries would have a distinct impact on the development of wheelchair services that promote social inclusion and independence.

## Abbreviations

ASBI: Adaptive Social Behaviour Inventory; ASSIA: Applied Social Sciences Index and Abstracts; BDI: Battelle Developmental Inventory; CASP: Critical Appraisal Skills Programme; CEBMa: Center for Evidence-Based Management; CI: Confidence Interval; COPM: Canadian Occupational Performance Measure; CINAHL: Cumulative Index to Nursing and Allied Health Literature; DARE: Database of Abstracts of Reviews of Effects; ECI: Early Coping Inventory; EPIOC: Electric powered indoor/outdoor chair; EPPI-centre: Evidence for Policy and Practice Information and Co-ordinating Centre; GMFM: Gross Motor Functional Measure; HTA: Health Technology Assessment; HUI: Health Utilities Index; ICIS: Impact of Childhood Illness Scale; MATCH: Matching Assistive Technology & Child; MEDLINE: Medical Literature Analysis and Retrieval System Online; MeSH: Medical Subject Headings; NFPO: Not for profit organisation; NHS: National Health Service; NHS EED: National Health Service Economic Evaluation Database; NICE: National Institute for Health and Clinical Excellence; PEDI: Pediatric Evaluation of Disability Inventory; PedsQL: Pediatric Quality of Life Inventory; PSSC: Parental Stress and Support Checklist; PIQ: Performance Intelligence Quotient; PKBS: Preschool and Kindergarten Behaviour Scales; PLS-3: Preschool Language Scale-3; PPVT: Peabody Picture Vocabulary Tests; PWC: Powered wheelchair; QALY: Quality adjusted life year; QUEST: Quebec User Evaluation of Satisfaction with assistive Technology; RCT: Randomised controlled trial; SF36: Short Form 36; SS: Sample size; UK: United Kingdom; VIQ: Verbal Intelligence Quotient; WHO: World Health Organisation; York CRD: University of York Centre for Research and Dissemination.

## Competing interests

The authors have no financial or non-financial competing interests to declare.

## Authors’ contributions

NB, JN, RTE and NH were responsible for the review conception and design and the development of the original review protocols. All authors were involved in reading and approving the manuscript. NB conducted the searches, quality appraisal, data extraction processes, analyses of evidence and synthesis of evidence, with support from JN, RTE and NH. JN provided evidence synthesis expertise and supervisory support.

## Authors’ information

NB is a Health Economics PhD student at Bangor University, supervised by RTE, JN and NH. RTE is Professor of Health Economics at Bangor University. She has expertise in the economic evaluation alongside trials of complex public health and psychosocial interventions, and an interest in the health economics of disability. JN has experience in child health research, health services research with embedded health economics and evidence synthesis. NH is Director of the Bath Institute of Medical Engineering (Designability) and a visiting Professor from University of Bath. His recent research projects include the use of assistive technology to support home based rehabilitation following stroke and monitoring patterns of physical activity during rehabilitation.

## Pre-publication history

The pre-publication history for this paper can be accessed here:

http://www.biomedcentral.com/1472-6963/14/309/prepub

## Supplementary Material

Additional file 1 **Included studies and reports.** Full list of included studies and reports, with details of study design, objectives, hypotheses, outcomes, measures, sample size, country, key findings and statistical evidence (where appropriate). Click here for file

Additional file 2 **Quality appraisal tools and outcomes.** Full list of quality appraisal tools and quality appraisal outcomes for each intervention, opinion and economic evidence study. Click here for file
