# Supplementary material for: Wheelchair interventions, services and provision for disabled children: a mixed-method systematic review and conceptual framework
Source: BMC Health Serv Res. 2014 Jul 17;14:309. doi: 10.1186/1472-6963-14-309 (PMC4110242; doi:10.1186/1472-6963-14-309)
Supplement: Additional file 1 — Included studies and reports. Full list of included studies and reports, with details of study design, objectives, hypotheses, outcomes, measures, sample size, country, key findings and statistical evidence (where appropriate). [file 1472-6963-14-309-S1.docx]

| **Author(s)**  Additional file 1: Included studies and reports  ***See full paper for references*** | **Study or document type; methodology*, aims / objectives / hypotheses*; outcomes / measures***  ****NA for reports*** | **Participants* / patient group; sample size (SS)*; country**  ****NA for reports*** | **Main findings (significant results for quantitative studies)** |
| --- | --- | --- | --- |
| All Party Parliamentary Group for Paediatric Wheelchair Reform and Whizz-Kidz (2011) [47] | Report | Young wheelchair users; UK | Aim to provide the right wheelchair, based on the right assessments, at the right time. Recommend changes to eligibility criteria to allow provision based on need; reducing expenses so cost of wheelchairs is not prohibitive; ensuring wheelchair services are high on the political agenda; clear and appropriate minimum standards; initial investment to kick-start changes; and joint working across local and national government departments. |
| Audit Commission (2002) [12] | Report | Wheelchair users; UK | Ineffectual service commissioning is a major cause of service issues and commissioners are not considering the vital contribution that wheelchair services provide to independence and reductions in morbidity. |
| Barnardos and Whizz-Kidz (2006) [43] | Report | Young wheelchair users; UK | Recommendations included reduced waiting times for assessment and delivery; ensure wheelchairs are suitable/have essential accessories, taking into account additional needs of carers/service users; listen to the needs and choices of children. |
| Benedict, et al (1999) [30] | Descriptive, non-parametric and qualitative procedures were used. Telephone survey and interviews used.  To determine the impact of assistive technology device use on child and family function and whether use by young children is related to caregiver satisfaction with a device.  **Measures:** Quebec User Evaluation of Satisfaction with assistive Technology- modified (QUEST); Pediatric Evaluation of Disability Inventory (PEDI) | Parents of a child whom a request for assistive technology device had been placed between child’s second and fourth birthday; 11 of 13 children diagnosed with cerebral palsy; Child age range: 34 to 54 months  SS=13; USA | No statistical analysis conducted. Regular manual wheelchair use in all places improved functional skill and reduced need for caregiver assistance. Evidence that the environment (manoeuvrability in the home) and the user (child’s interest in device) made devices difficult to use regularly and effectively. Carer statements indicated that wheelchairs offered increased independence and reduced need for assistance. |
| Bottos et al (2001) [25] | Quasi-experimental trial  To investigate the effects of early PWC provision on objective outcome indices and subjective outcome indexes. To see which individuals could achieve a good-enough driving competence and what conditions would influence the achievement/non-achievement of this competence.  **Measures:** Performance Intelligence Quotient (PIQ) score of the Leiter International Performance Scale; Verbal Intelligence Quotient (VIQ) score of the Peabody; Developmental Verbal Scale; Gross Motor Functional Measure (GMFM); Canadian Occupational Performance Measure (COPM); Furumasu's Driving Test; Parental perceptions; Impact of Childhood Illness Scale (ICIS) | Children diagnosed with cerebral palsy; Age range: 3 to 8 years  SS=25; Italy | PWC provision had significant positive effects to activities of daily life (in the dimension of functional limitation) (p<0.00001), satisfaction with performance (p<0.00001) and PWC driving competence after 6 to 8 months of use (p<0.01). Confidence intervals not reported. |
| Care Services Improvement Partnership (2006) [44] | Report | Wheelchair users; UK | Commissioners, in partnership with providers, need to ensure that children and carers are involved in decisions about their care; that wheelchair provision is based on individual need; that transition from child to adult services is planned for; that wheelchairs are provided promptly based on multiagency assessment; that service users are provided with wheelchairs that can ‘grow’ to meet their changing needs; and that there is a reasonable balance between risk of denying a wheelchair and the safety of a supplied wheelchair. |
| Curtin and Clarke (2005) [40] | Biographical research  To investigate the life stories of a small number of young people with physical disabilities, in particular focusing on their educational experiences.  **Measures:** Qualitative interviews | Children using manual or powered wheelchairs; Age range: 10 to 13 years  SS=9; UK | Physical disability and wheelchair use had an effect on the choice of school a child attends (mainstream or segregated special school) based on facilities, level of assistance/support and attitudes of other pupils. |
| Deitz et al (2002) [26] | Single-subject withdrawal design  To explore the effects of a powered mobility riding toy on the participation behaviours of young children with complex developmental delays.  **Measures:** Child-initiated movement; Initiation of contact with others; Affect (positive, neutral and negative facial expression). | Young children with complex developmental delays; Age range: 4 to 5 years  SS=2; USA | No statistical analysis conducted. Use of a powered riding toy had positive impact on initiation of movement occurrences. |
| Department of Health (2004) [16] | Report | Disabled children and those with complex healthcare needs; UK | Disabled children should receive coordinated, high-quality, child and family centred services and these services should promote social inclusion. Appropriate housing and assistive mobility technology is essential and should be promoted by facilitating families with disabled children to live in suitable housing and have the appropriate assistive mobility technology to promote the wellbeing of disabled children and their families. Integration across services that supports personalised, child-centred care to every disabled child. |
| Department of Health Commissioning Team (2010) [9] | Report | Wheelchair users; UK | Key issues identified included focusing on the needs of the user; achieving timely access; ensuring equity of provision; improving outcomes for service users; adopting a preventative approach to service provision; shifting balance of resources from service management to wheelchair provision; and encouraging innovation. |
| Durkin (2009) [32] | Qualitative Grounded Theory  To explore the question- "How does a child learn to use powered mobility to explore their environment".  **Measures:** Qualitative interviews | Children with disabilities using powered wheelchairs and peer professionals; Age range: 5-12  SS= Typically developing children: 11; Powered mobility users: 18; Peer professionals: 22; UK | Wheelchairs can be more than a tool for movement: a source of enjoyment through play and games, as well as a tool for understanding movement and maintaining development. Playing and driving to learn are important. Parental input was best as a ‘responsive partner’ facilitating learning. |
| Evans et al (2007) [7] | Qualitative analysis- a priori interviews  To examine the experiences of severely physically disabled young people using electric powered indoor/outdoor chairs (EPIOCs). To address the gap in the young EPIOC user literature by undertaking a qualitative analysis of young people's experience using NHS supplied EPIOC.  **Measures:** Qualitative interviews | Young people with physical disabilities using manual and powered wheelchairs; Age range: <18 years.  SS=18; UK | NHS wheelchair services generally considered satisfactory and helpful, although waiting times were an issue. Use of EPIOC was considered positive and beneficial for development and independence. More training was wanted to help limit accidents. Bulkiness and difficulty of transporting were issues. |
| Frontier Economics (2011) [52] | An analysis of Whizz-Kidz work with a primary care trust to improve wheelchair provision.  To examine the quantitative impacts of Whizz-Kidz' work with the NHS on children accessing these services.  **Measures:** Cost per QALY of meeting additional unmet demand. Secondary sources of utility data | Children with physical disabilities using wheelchairs for mobility; Age range: not stated.  SS=not stated; UK | Meeting unmet demand by Whizz-Kidz cost an extra £108,000 and provided an additional 10.7 to 14 QALYs. This resulted in a cost per QALY of between £7,700 and £9,800 to meet additional unmet demand. |
| Furumasu et al (2008) [22] | Case series  To provide more objective evidence of the benefits of powered mobility for young children to aid clinicians in justifying a PWC recommendation to families and physicians.  **Measures:** Adaptive Social Behaviour Inventory (ASBI); Preschool and Kindergarten Behaviour Scales (PKBS); Peabody Picture Vocabulary Tests (PPVT-IIIA); Preschool Language Scale-3 (PLS-3); Symbolic Play Assessment; Survey of Technology Use. | Children with orthopaedic disabilities (non-cerebral palsy) and children with cerebral palsy; Age range: 18 to 72 months.  SS=23; USA | Significant positive effects after PWC provision to pro-social adaptive social behaviour (F=5.30, p<.05 at 95%CI); interactions with family (F=3.2, p<.05 at 95%CI); indoor play motor activities (F=4.53, p<.05 at 95%CI); quality of interactive play (F=4.24, p<.05 at 95%CI); and developmental level of symbolic play (F=4.9, p<.05 at 95%CI). |
| Guerette et al (2005) [33] | Questionnaire Survey  To identify the most common reasons for not recommending a powered wheelchair, and the reasons why a child who is recommended a PWC does not receive one. To gather information regarding the current practice used to evaluate a child for a PWC and the typical recommendation for children who are not prescribed a PWC. To obtain objective and subjective baseline data regarding the impact of PWC on young children.  **Measures:** Qualitative interviews | Suppliers and Clinicians; Child age range: 2 to 6 years  SS=140; USA | Cognitive factors and behavioural factors were considered (by clinicians and suppliers) the most common cause for non-recommendation of PWC, while funding issues and lack of family support were considered most common reasons why a PWC was not received. |
| HM Treasury and Department for Education and Skills (2007) [45] | Report | Children and young people with disabilities; UK | Government should take action in three priority areas to improve outcomes for disabled children: access and empowerment; responsive services and timely support; improving quality and capacity. |
| Home and Ham (2003) [34] | Retrospective survey  Exploring the experiences of children issued with powered mobility by the charity Whizz-Kidz.  **Measures:** questionnaire survey | Families of children who had been issued with a PWC from Whizz-Kidz between 1990 and 2000; Child age range: <7 years.  SS=57; UK | General consensus on a number of positive effects of PWC use including independent mobility and facilitation of integration with other children. Several issues were highlighted, including costs of maintenance and suitability of wheelchairs. |
| Huhn et al (2007) [27] | Case report/description  To describe the physical therapist's clinical decision making related to power mobility for a child with multiple disabilities.  **Measures:** Driving wheelchair through a standard width doorway; Manoeuvring through three cones set three feet apart; Driving wheelchair through a 100-foot hallway with typical school traffic. | Children with spastic quadriplegic cerebral palsy; Age range: 9 to 12 years.  SS=1; USA | No statistical analysis conducted. For disabled children with multiple impairments mid-wheel drive PWCs facilitate better control as compared with rear-wheel drive equivalent PWCs. Results indicated that length of time training and learning to use a PWC correlated with achieving independence. Motivation and level of frustration impact length of time taken to achieve independence. |
| Jones et al (2003) [21] | Case report  To show that a child as young as 20 months of age can learn to use a PWC. To describe the procedures used in training a young child to use a PWC and in evaluating the developmental changes made after receiving the PWC.  **Measures:** Powered mobility skills- 7 question powered mobility skills indicator; Battelle Developmental Inventory (BDI); PEDI. | Child with spinal muscular atrophy; Age range: 17 to 23 months.  SS=1; USA | No statistical analysis conducted. Communicative, personal-social and cognitive development increased by greater than the expected age-equivalent scores (for typically developing peers) after provision of PWC. Positive trends in self-care, mobility and social function after intervention. |
| Jones et al (2012) [23] | Randomised controlled trial  To identify any effects of PWC on the development and function of young children with severe motor impairments.  **Measures:** BDI; PEDI; Early Coping Inventory (ECI) | Children with motor impairment preventing functional independent mobility; Age range: 14 to 30 months.  SS=14 (control), 14 (intervention); USA | Significant improvements after PWC intervention (compared to control) in receptive communication (p=.03, Effect size=6.1 [0.95-9.2] at 90% CI); mobility functional skill (p=.04, Effect size=6.5 [2-11] at 90%CI); need for caregiver assistance for mobility (p=.01, Effect size=12.35 [6.5-20.5] at 90%CI); need for caregiver assistance for self-care (p=.0007, Effect size=11.95 [7.5-16.15] at 90%CI); and overall development scores (p=.083, Effect size=2.0 [0.0-3.5] at 90%CI). |
| Lawlor et al (2006) [38] | Qualitative in-depth interviews  To ascertain from families of children with cerebral palsy the features of social, attitudinal and physical environments which facilitate or restrict participation. **Measures:** Qualitative analysis | Families of children with cerebral palsy; Child age range: 4 to 17 years  SS=12; USA | PWC perceived to reduce the level of support required from parents/carers. Lack of space, ramps and adequate paths restricted access and leisure activities. Public buildings, toilets, transport and shopping aisles restricted participation. Barriers were costs, effects on earnings, negative attitudes of public and lack of knowledge regarding benefits. Wheelchair services act as barriers to participation due to long waiting times for wheelchairs. |
| Meiser and McEwen (2007) [28] | ABA Single Subject design  To compare propulsion in young children with spina bifida when using two styles of wheelchairs: an ultralight titanium rigid-frame wheelchair and a lightweight aluminium folding frame wheelchair.  **Measures:** Speed for 50ft in controlled environment; speed in a typical school activity; moving in the hallway as the line-leader of classmate; distance of propulsion in familiar surroundings; energy expenditure; energy expenditure index; perceived exertion. | Children with spina bifida (L2 or above); Age range: <6 years.  SS=2; USA | No statistical analysis conducted. Ultralight wheelchairs facilitated better results than lightweight wheelchairs in propulsion. Both parents and children indicated preference for the ultralight chairs over lightweight chairs. |
| Muscular Dystrophy Campaign (2010) [41] | Report | Wheelchair users; UK | Waiting times for wheelchairs should be a maximum of 18 weeks from initial referral to delivery. A national consensus of a uniform eligibility criterion is required to improve equality in wheelchair provision criterion should be established to improve equity in services. Maintenance of wheelchairs (including privately funded chairs) should be covered by NHS primary care trusts. |
| Muscular Dystrophy Campaign (2011) [42] | Report | Wheelchair users with muscular dystrophy and other neuromuscular conditions; UK | Key identified issues included supplying wheelchairs that promote independence; support good posture; allow exploration and opportunities for play; promote development; and restrict development of deformities. |
| National Assembly for Wales Health, Wellbeing and Local Government Committee (2010) [11] | Report | Wheelchair users; UK | Recommendations included defining a strategic plan to promote better integration with other services and organisations; performance measures focussing on outcomes for users; better provision of information to users; streamlining of referrals process; exploring the possibility of pooling existing budgets; reviewing arrangements for short-term loans of wheelchairs; and ensuring that regular reviews for service users (especially those with changing needs) are maintained. |
| Neilson et al (2000) [48] | Case series / cost-effectiveness analysis  The present exploratory study considered the impact of a wide range of surgical and orthotic interventions on the quality of life of children and adults with profound intellectual and multiple disabilities.  **Measures:** Short Form 36 (SF36); QALY gains | Children and adults with profound intellectual and multiple disabilities (22=cerebral palsy); Age range: 2 to 55 years (mean age= 19).  SS=1 relevant to review; Scotland | Cost per QALY (compared with a ‘do nothing’ scenario) for provision of an EPIOC ranged from £734 to £1378 (dependent on time horizon) based on a cost per wheelchair intervention ranging from £1500 to £2000. |
| NHS Modernisation Agency (2005) [13] | Report | Wheelchair users; UK | Recommendations included agreed eligibility criteria and information/tuition for each chair supplied; efficient use of resources and minimising delay; 100% of standard prescriptions delivered within 10days; and all users made aware of mechanism for contact/review. |
| Østensjø et al (2005) [29] | Cross-sectional  To describe all assistive devices and other environmental modifications provided to support everyday activities in young children with cerebral palsy, and the benefits of these modifications for functioning and care giving.  **Measures:** PEDI; Classification of Technical Aids for Persons with Disabilities; Questionnaire- sociodemographic factors and associated problems. | Children with cerebral palsy and their parents; Age range: 2 to 7.5 years.  SS=95; Norway | Strong correlation between the amount of caregiver assistance and the number of modifications in use for purposes of mobility (p<0.001) and level of independence and the demands placed on caregivers (p=0.002 to <0.001). Confidence intervals not reported. |
| Prime Minister's Strategy Unit (2005) [14] | Report | People with disabilities; UK | Government policy should enable young disabled children and their families to live ‘ordinary’ lives, through effective support in mainstream settings. There should be timely access to wheelchairs and a specific key worker for each family with high needs. Supply based on a multi-agency assessment and should be delivered promptly after assessment. Strategic planning between health, social services and education. |
| Scottish Executive (2006) [46] | Report | Wheelchair users; Scotland | Recommendations included comprehensive access to multi-disciplinary teams for assessment and review; all services undertaken in child-oriented facilities; clinics providing access to specialist paediatric clinical expertise; access to extended wheelchair loan programmes; and the establishment of properly functioning multi-agency links. |
| Shahid (2004) [39] | Questionnaire Survey  To explore the opinions between health professionals and parents of children with cerebral palsy with regard to factors affecting buggy-to-wheelchair progression.  **Measures:** Questionnaire survey | Parents of children with cerebral palsy and healthcare professionals; Child age range: 3 to 14 years.  SS= Parents: 28  Professionals: 17; UK | Most common issues preventing buggy-to-wheelchair progression were transport problems (private and public); housing problems inhibiting storage and mobilisation of wheelchair; lack of information regarding positives of progression; and carer difficulty in manoeuvring a wheelchair. There were difference in opinions between professionals and parents. |
| Staincliffe (2003) [35] | Questionnaire survey  To find out the current policy and practice for issuing PWC to children.  **Measures:** Questionnaire survey | NHS wheelchair services; Child age range: not stated.  SS=69; UK | 54% of NHS wheelchair services receive referrals for PWC for children under the age of 5, 10% had an age exclusion policy (children less than 5 years old not eligible for PWC). |
| Tefft et al (2011) [24] | Questionnaire survey  To evaluate the impact of PWC on parental stress, negative emotions, perceived social interactions and parental satisfaction with wheelchair characteristics such as size and durability.  **Measures:** Developmental Observation Checklist System Part III Parental Stress and Support Checklist (PSSC); Matching Assistive Technology & Child (MATCH); Survey of Technology Use; QUEST | Parents of children with orthopaedic disabilities or cerebral palsy; Child age range: 18 to 42 months (children with orthopaedic disabilities), 18 to 72 months (children with cerebral palsy).  SS=23; USA | PWC provision facilitated significant improvement in parents’ satisfaction with child’s ability to go where they desire (*F*[2,21]=11.69, p<.05); interactions with family (*F*[2,21]=3.3, p<.05); parental satisfaction with child’s social and play skills (*F*[2,21]=3.27, p<.05); and parents’ belief that the general public accepts their child (*F*[2,21]=3.65, p<.04). |
| Welsh Assembly Government (2005) [8] | Report | Children and young people (with and without disabilities); Wales | Disabled children should have access to any assistive mobility technology they require, wherever they require it, and services should be integrated across health, social care and education, to enable multiagency assessments and streamline of provision. Key priorities include prompt services; in depth assessments; family support services; educational support services; clear and accurate information; and emotional support services. |
| Wiart et al (2003) [36] | Telephone survey/interview  To explore environmental (i.e. physical, social and attitudinal) barriers and facilitators to successful powered mobility use with the participants and their families.  **Measures:** Questionnaire survey | Young people who had received a PWC at age 18 or under; Age range: 4.5 to 27.5 years (mean= 15.2 years).  SS=66; Canada | Reasons why PWC use was discontinued included concerns regarding safety; poor fit between type of PWC/access system and user; change in medical/functional abilities; and lack of success with using PWC effectively. Barriers to PWC use included physical barriers at public and private buildings and transportation issues. |
| Wiart et al (2004) [31] | Phenomenology  To explore parents' experiences and perceptions of their children's experiences with powered mobility.  **Measures:** Qualitative interviews | Mothers of children with physical disabilities using powered mobility devices; Child age range: 10 to 18 years.  SS=5; Canada | Consensus that PWC use can facilitate a number of benefits including independence; participation in social and peer age-appropriate activities; facilitation meaningful relationships; and development of personal control. Initial attitude to both PWC and manual chairs was of sadness and despair, which after PWC provision developed into positive feelings about the benefits of PWC. |
