# Supplementary material for: Wheelchair interventions, services and provision for disabled children: a mixed-method systematic review and conceptual framework
Source: BMC Health Serv Res. 2014 Jul 17;14:309. doi: 10.1186/1472-6963-14-309 (PMC4110242; doi:10.1186/1472-6963-14-309)
Supplement: Additional file 2 — Quality appraisal tools and outcomes. Full list of quality appraisal tools and quality appraisal outcomes for each intervention, opinion and economic evidence study. [file 1472-6963-14-309-S2.docx]

# Additional File 2: Quality Appraisal Tools and Outcomes

**Key**

Y-Yes

N- No

NC- Not clear

**Supplementary Table 1: Qualitative Study Appraisal Outcomes [1a]**

|  | **Question** | | | | | | | | | |
| --- | --- | --- | --- | --- | --- | --- | --- | --- | --- | --- |
| **Paper** | **1** | **2** | **3** | **4** | **5** | **6** | **7** | **8** | **9** | **10** |
| Evans et al, 2007 [7] | Y | Y | Y | Y | NC | N | Y | NC | Y | High Value |
| Lawlor et al, 2006 [38] | Y | Y | Y | NC | Y | N | Y | NC | Y | High Value |
| Curtin & Clarke, 2005 [40] | Y | Y | Y | NC | Y | N | Y | NC | Y | High Value |
| Wiart et al, 2004 [31] | Y | Y | Y | NC | Y | N | Y | Y | Y | High Value |
| Durkin, 2009 [32] | Y | Y | Y | NC | Y | Y | Y | Y | Y | High Value |

**Supplementary Table 2: Questionnaire Survey Appraisal Outcomes [2a]**

|  | **Question** | | | | | | | | | | | |
| --- | --- | --- | --- | --- | --- | --- | --- | --- | --- | --- | --- | --- |
| **Paper** | **1** | **2** | **3** | **4** | **5** | **6** | **7** | **8** | **9** | **10** | **11** | **12** |
| Guerette et al, 2005 [33] | Y | Y | Y | NC | NC | Y | Y | NC | Y | N | NC | Y |
| Shahid, 2004 [39] | Y | Y | Y | NC | Y | N | NC | NC | N | N | Y | Y |
| Home & Ham, 2003 [34] | Y | Y | Y | N | NC | N | N | NC | N | N | Y | Y |
| Staincliffe, 2003 [35] | Y | Y | Y | N | Y | N | NC | NC | N | N | NC | Y |
| Wiart et al, 2003 [36] | Y | Y | Y | NC | NC | N | Y | NC | N | N | NC | Y |
| Tefft et al, 2011 [24] | Y | Y | Y | NC | NC | N | Y | Y | Y | Y | NC | Y |
| Benedict et al 1999 [30] | Y | Y | Y | N | NC | N | Y | Y | N | N | N | Y |

**Supplementary Table 3: Case Study Appraisal Outcomes [3a]**

|  | **Question** | | | | | | | | | |
| --- | --- | --- | --- | --- | --- | --- | --- | --- | --- | --- |
| **Paper** | **1** | **2** | **3** | **4** | **5** | **6** | **7** | **8** | **9** | **10** |
| Huhn et al, 2007 [27] | Y | Y | NC | N | Y | N | N | N | NC | NC |
| Jones et al, 2003 [21] | Y | Y | NC | N | Y | N | N | NC | NC | NC |

**Supplementary Table 4: Descriptive / Cross-Sectional Study Appraisal Outcomes [4a]**

|  | **Question** | | | | | | | | | | |
| --- | --- | --- | --- | --- | --- | --- | --- | --- | --- | --- | --- |
| **Paper** | **1** | **2** | **3** | **4** | **5** | **6** | **7** | **8** | **9** | **10** | **11** |
| Østensjø et al, 2005 [29] | Y | Y | NC | Y | NC | NC | Y | Y | Y | NC | Moderate Value |

**Supplementary Table 5: Randomised Controlled Trial Appraisal Outcomes [5a]**

|  | **Question** | | | | | | | | | |
| --- | --- | --- | --- | --- | --- | --- | --- | --- | --- | --- |
| **Paper** | **1** | **2** | **3** | **4** | **5** | **6** | **7** | **8** | **9** | **10** |
| Jones et al, 2012 [23] | Y | Y | Y | Y | Y | Y | NC | Appropriate presentation | High accuracy | NC |

**Supplementary Table 6: Quasi-Experimental Study Appraisal Outcomes [6a]**

|  | **Question** | | | | | | | | | | |
| --- | --- | --- | --- | --- | --- | --- | --- | --- | --- | --- | --- |
| **Paper** | **1a** | **1b** | **2a** | **2b** | **2c** | **3a** | **3b** | **4a** | **4b** | **5a** | **5b** |
| Bottos et al, 2001 [25] | Y | NC | Y | Y | N | Y | Y | Y | N | NC | NC |
| Meiser & McEwan, 2007 [28] | Y | Y | Y | Y | N | NC | NC | Y | N | NC | NC |
| Furumasu et al, 2008 [22] | Y | Y | NC | Y | Y | Y | Y | NC | N | NC | Y |

**Supplementary Table 7: Economic Evaluation Appraisal Outcomes [7a]**

|  | **Question** | | | | | | | | | | | |
| --- | --- | --- | --- | --- | --- | --- | --- | --- | --- | --- | --- | --- |
| **Paper** | **1** | **2** | **3** | **4a** | **4b** | **4c** | **5** | **6** | **7** | **8** | **9** | **10** |
| Neilson et al, 2000 [48] | Y | Y | NC | Y | Y | NC | Y | NC | NC | N | Y | Y |
| Frontier Economics, 2010 [52] | Y | NC | NC | N | NC | NC | NC | Y | NC | NC | NC | Y |

**Additional File 1 Supplementary References**

1a. Critical Appraisal Skills Programme (CASP): **Qualitative Study CASP Tool** [http://www.sph.nhs.uk/what-we-do/public-health-workforce/resources/critical-appraisals-skills-programme]

2a. Centre for Evidence Based Management (CEBMa): **Questionnaire Survey CEBMa Tool** [http://www.cebma.org/ebp-tools/]

3a. Centre for Evidence Based Management (CEBMa): **Case Study CEBMa Tool** [http://www.cebma.org/ebp-tools/]

4a. Milton Keynes Primary Care Trust: **11 questions to help you make sense of descriptive/cross-sectional studies [**<http://reache.files.wordpress.com/2010/03/cross-sectional-appraisal-tool.pdf>]

5a. Critical Appraisal Skills Programme (CASP): **Randomised Controlled Trial CASP Tool** [http://www.sph.nhs.uk/what-we-do/public-health-workforce/resources/critical-appraisals-skills-programme]

6a. Greenhalgh T, Robert G, Bate P, Macfarlane F, Kyriakidou O: *Diffusion of Innovations in Health Service Organisations: A systematic literature review*. Oxford: Blackwell Publishing, BMJ Books [http://onlinelibrary.wiley.com/doi/10.1002/9780470987407.app2/pdf]

7a. Critical Appraisal Skills Programme (CASP): **Economic Evaluation CASP tool** [http://www.sph.nhs.uk/what-we-do/public-health-workforce/resources/critical-appraisals-skills-programme]
